# Understanding responses of people with ASD in diverse reasoning tasks: A formal study

**DOI:** 10.1007/s10339-024-01233-w

**Published:** 2024-10-07

**Authors:** Torben Braüner, Aishwarya Ghosh, Sujata Ghosh

**Affiliations:** 1 Roskilde University, Universitetsvej 1, 4000 Roskilde, Denmark; 2 University of Utah, 260 Central Campus drive, Salt Lake City, UT 84112 USA; 3 Indian Statistical Institute, Aminjikarai, Chennai, Tamil Nadu 600029 India

**Keywords:** Autism spectrum disorder, Syllogisms, Decision task, Context stimuli, The attraction effect, The sunk-cost effect, The Linda task

## Abstract

Recent studies have shown that in some reasoning tasks people with Autism Spectrum Disorder perform better than typically developing people. This paper compares four such tasks, namely a syllogistic task, two decision-making tasks, and a task from the heuristics and biases literature, the aim being to identify common structure as well as differences. In the terminology of David Marr’s three levels of cognitive systems, the tasks show commonalities on the computational level in terms of the effect of contextual stimuli, though an in-depth analysis of such contexts provides certain distinguishing features in the algorithmic level. We also make some general remarks on our approach, so as to set the stage for further studies in the area which could provide a better understanding of the reasoning process of ASD individuals.

## Introduction

It is well-known from the vast psychological and psychiatric literature on Autism Spectrum Disorder (ASD),^1^ that children with ASD have a limited or delayed capacity to respond correctly to psychological reasoning tests called false-belief tasks. In other words, on such tests, ASD children perform less well than children with a typical development (TD). However, it turns out that in some other reasoning tasks, ASD people performs not *worse*, but *better*, than typicals, thus, showing that ASD is not in all respects a “disability”, a view that was put forward by Baron-Cohen (2000), two decades ago. During the last few years, several new empirical studies have emerged where ASDs perform better than typicals, thus supporting Baron-Cohen’s view.

Farmer et al. (2017) investigate adult’s performance in a decision task where the subject has to choose between pairs of consumer products that are presented with a third, less desirable “decoy” product. According to conventional economic theory, a consumer’s choice of one product over another should be independent of whether there is a third option. To quote the paper, “If one prefers salmon to steak, this should not change just because frogs’ legs are added to the menu”. Farmer et al. demonstrate that the tendency to violate this norm is reduced among individuals with ASD, thus, in this sense, they are more rational than typical individuals. They found a similar difference between the two groups of people drawn from the general population, classified in accordance with their levels of autistic-like traits, measured in terms of the self-report questionnaire called the Autism-Spectrum Quotient (AQ).

From a probabilistic reasoning perspective, Morsanyi et al. (2010) compare the performance of ASD and typical adolescents on tasks from the heuristics and biases literature, including a task called the conjunction fallacy; a fallacy that involves violating a fundamental law of probability theory. It is found that adolescents with ASD are less susceptable to this fallacy than typicals. Two explanations are considered: (i) autistic people are more sensitive to probabilistic information, that is, they are more logical in a normative sense, and (ii) autistic people are less sensitive to irrelevant contextual information. In fact, the study in Morsanyi et al. (2010) includes control tasks, allowing a direct comparison between ASDs and typical individuals in terms of their capacity to observe probability rules, leading to the second explanation.

An example from logic can be found in Lewton et al. (2019), where Lewton et al. compare the ability to do syllogistic reasoning in the general population with individuals showing autistic-like traits, measured in terms of the AQ-score. A syllogism is a logical reasoning pattern with two premises and one conclusion. If the conclusion can be logically deduced from the premises, the syllogism is said to be valid, otherwise it is said to be invalid. Some syllogisms are consistent with reality: *All birds have feathers. Robins are birds. Therefore robins have feathers*, but others are not: *All mammals walk. Whales are mammals. Therefore whales walk.* Both these syllogisms are valid, in fact, they have exactly the same logical structure, but the validity is more difficult to detect in the second syllogism because the correct answer is inconsistent with reality. In other words, people are more prone to make reasoning errors when the conclusion disagrees with prior knowledge. The study in Lewton et al. (2019) shows that there is a negative correlation between this reasoning bias and the AQ-score, thus, the more autistic-like a person is, the better the person is to judge syllogisms without being affected by irrelevant prior knowledge of reality. We suggest the readers to see Khemlani and Johnson-Laird (2012) for a comprehensive overview of different psychological theories of syllogistic reasoning.

Fujino et al. (2019) investigate adults’ performance in the so-called sunk cost task which measures the tendency to include considerations on past costs when choosing between current alternatives, for example, when continuing an expensive investment even though higher costs, rather than benefits, are expected in the future. According to conventional economical theory, past expenses are irrelevant, rational decision makers should only pay attention to future consequences of possible alternatives. The empirical study reported in Fujino et al. (2019) shows that the people with ASD are less prone to violate this norm than the typical individuals.

Now, to the best of our knowledge, there are no theoretical and formal studies of the commonalities between the psychological tasks where individuals with ASD perform better than the typical individuals,^2^ as reported in Farmer et al. (2017); Lewton et al. (2019); Fujino et al. (2019); Morsanyi et al. (2010). It is the goal of the present paper to investigate this question—an interdisciplinary enterprise requiring insights from both logic and economic theory. Such an investigation will help us in providing a better understanding of the capabilities of the individuals with ASD, which in turn might help in accommodating a better work environment for these individuals. A common feature of the above-mentioned tasks seems to be that they require an ability to disregard irrelevant contextual information, but this is a very informal verbal description. In what follows, we will provide a survey of the studies mentioned above towards providing a more formal analysis in an attempt to find a common structure, inspired by other works aiming at identifying a common logical structure in superficially different reasoning tasks.^3^

As a tool to analyse the tasks in question, we shall make use of David Marr’s levels of analysis of cognitive systems: Marr (1982) has influentially argued that any task computed by a cognitive system must be analyzed at the following three levels of explanation (in order of decreasing abstraction):

Computational level: Identification of the goal and of the information-processing task as an input–output function;
Algorithmic level: Specification of an algorithm which computes the function;
Implementational level: Physical or neural implementation of the algorithm.

**Fig. 1:**
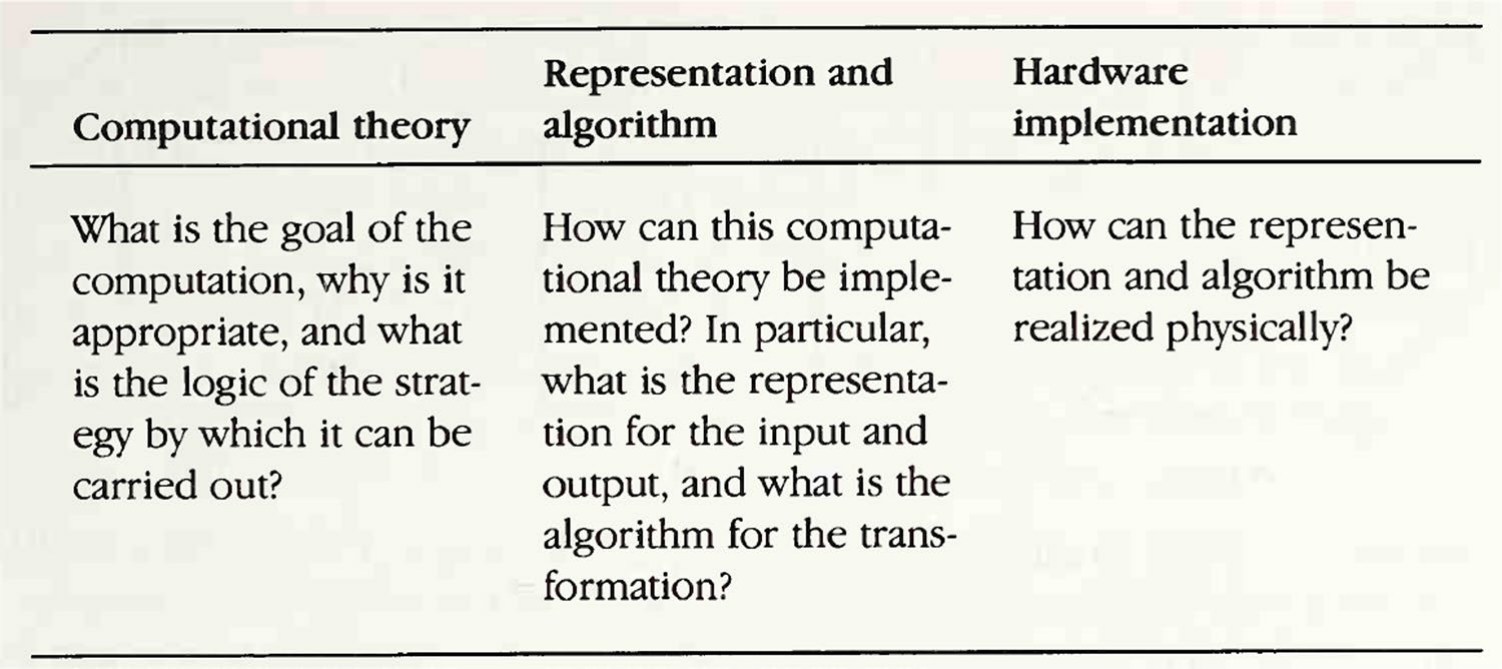
Marr’s levels, taken from Marr (1982)

See also Fig. 1 for an explication of Marr’s levels. Analogous levels of analysis can be found in several other works of cognitive science, e.g., see the overview in Stanovich (1999), pages 9–12. For this work, we shall focus on the computational and algorithmic levels.

The remainder of the paper can be described as follows: The four kinds of tasks described above are presented in the next four sections together with their computational and algorithmic analyses. Section 6 discusses the commonalities and differences among these tasks with respect to the behavior of the ASD individuals in these tasks. Section 7 concludes the paper.

## The syllogistic task

In this section, we analyze the performances in the syllogistic tasks as investigated in Lewton et al. (2019) on both computational and algorithmic levels. We first provide a brief discussion on the empirical study as reported in Lewton et al. (2019).

**An empirical study by **Lewton et al. (2019): Four different types of syllogisms are considered. The two syllogisms described in the introduction were of the respective types of valid-believable and valid-unbelievable (recall that a syllogism is valid if the truth of the premises implies the truth of the conclusion and a syllogism is believable if the conclusion is actually true). But there are also the types invalid-believable and invalid-unbelievable. An example of the former type is: *All flowers need water. Roses need water. Therefore roses are flowers*. An invalid-unbelievable syllogism with exactly the same structure is: *All insects need oxygen. Mice need oxygen. Therefore mice are insects*. The four different types of syllogisms are summed up in Table 1.

In Lewton et al. (2019), each subject has to judge four congruent syllogisms (valid-believable and invalid-unbelievable) and four incongruent ones (invalid-believable and valid-unbelievable). A subject scores 1 point for each correct judgement. So there is a 0–4 scale for congruent syllogisms and 0–4 for incongruent ones. A belief bias occurred when there is a decrease in accuracy for incongruent problems (valid-unbelievable and believable-invalid) relative to congruent problems (valid-believable, invalid-unbelievable). Such a bias is calculated by subtracting the score for incongruent syllogisms from that of congruent ones, resulting in a possible score from $-$ 4 to 4. The study reports a number of correlation results, (controlled for sex since sex differences have been reported in the AQ test) as follows:

**Table:** 

CONCEPTS	AQ
Belief bias	$-$ 0.39 (*p* < 0.001)
Congruent syllogism	0.11 (Not significant)
Incongruent syllogism	0.40 (*p* < 0.001)

In addition, the congruent-incongruent correlation was $-\,0.04$ but not significant. Thus, it seems that the congruent and incongruent variables measure different underlying cognitive abilities, and only the latter is associated with AQ.

Besides the above study, we are only aware of one other published study relating performance on belief bias syllogisms to autistic-like traits, namely Lewton (2018), also by Lewton. On the other hand, typically developing individuals’ performance on belief bias syllogisms have been investigated in many studies, and has been found to correlate with a number of other measures of rational thinking, see for example the recent paper Young et al. (2018). See also the paper Klauer et al. (2000), which we shall come back to in Sect. 2.2.

### Computational level analysis (syllogistic task)

We now ask the following question: What does it more precisely mean that a subject is able to judge a syllogism without bias, that is, without involving irrelevant contextual information? First, a syllogism *If* $\phi _1$ *and* $\phi _2$, *then* $\psi$ is valid if and only if for any model ${{\mathcal {M}}}$, it is the case that if $\phi _1$ holds in ${{\mathcal {M}}}$ and $\phi _2$ holds in ${{\mathcal {M}}}$, then $\psi$ holds in ${{\mathcal {M}}}$ This defines a mathematical function ${ valid}$ which maps syllogisms to truth-values, that is, elements in the set $\{0,1\}$. For example, if *S* is one of the syllogisms in the upper row of Table 1, then ${ valid}(S)=1$, and if *S* is one of the syllogisms in the lower row of the table, then ${ valid}(S)=0$. Thus, the function ${ valid}$ formalize the normatively correct judgement of syllogisms.

**Table 1:** Example syllogisms

	Believable	Unbelievable
Valid	All birds have feathers Robins are birds ———————————— Therefore robins have feathers	All mammals walk Whales are mammals ————————— Therefore whales walk
Invalid	All flowers need water Roses need water ——————————— Therefore roses are flowers	All insects need oxygen Mice need oxygen ——————————– Therefore mice are insects

Now, an individual subject’s judgement of a syllogism takes place in a specific context, that is, in a specific state of affairs, namely the actual state of affairs, where for example *Robins have feathers* is true, but *Whales walk* is false. Such a state of affairs is formalized by a model. This means that a fixed subject’s judgement of syllogisms in a context can be modeled by a mathematical function ${ believable}$ similar to the function ${ valid}$, but with an extra parameter, representing a context. So if the actual state of affairs is formalized by ${{\mathcal {M}}}$, then ${ believable}(S, {{\mathcal {M}}})$ is the given subject’s response to a particular syllogism *S*. Thus, the function ${ believable}$ maps a pair consisting of a syllogism and a model to a truth-value, and the requirement of context-independence can be formulated as

1 $$\begin{aligned} { believable}(S, {{\mathcal {M}}}_1)={ believable}(S, {{\mathcal {M}}}_2) \end{aligned}$$

for any syllogism *S* and any models ${{\mathcal {M}}}_1$ and ${{\mathcal {M}}}_2$. A stronger requirement than the independence of context is the notion of correctness, that is,

2 $$\begin{aligned} { believable}(S, {{\mathcal {M}}})={ valid}(S) \end{aligned}$$

for any syllogism *S* and any model ${{\mathcal {M}}}$. Note that this is a strictly stronger requirement, for example, a ${ believable}$ function that always gives the incorrect answer would be independent of contexts. We note that whereas there is a correctness requirement for the conjunction task discussed in the next section, we did not find something similar to the notion of correctness in the cases of the decision task and the sunk cost task we discuss later. For example, if *S* is the valid and believable syllogism in the upper-left of Table 1, and ${{\mathcal {M}}}$ formalizes the real world (where, for example, robins have feathers), then

$$\begin{aligned} { valid}(S)=1 \hspace{5mm} \hbox { and } \hspace{5mm} { believable}(S, {{\mathcal {M}}})=1 \end{aligned}$$

Similarly, the remaining three syllogisms in the table yields the remaining three combinations of truth-values.

### Algorithmic level analysis (syllogistic task)

In what follows we shall describe a selection of theoretical explanations of belief bias in syllogistic reasoning, cf. the paper (Klauer et al. 2000). The explanations have the form of algorithms, where bias arises at one of three different stages in the reasoning process: during input, processing, or output, cf. Klauer et al. (2000), page 852. Given the algorithmic character of the explanations, we are situated at the second of Marr’s three levels, where an algorithm computes the input–output function specified at the top level. We give particular attention on the reasoning process that takes place when incongruent syllogisms are judged, that is, when logic and belief conflict.

A very simple algorithm described by Klauer et al. (2000) is called the *selective scrutiny model*, which works as follows: If the conclusion is believable, the algorithm just accepts that the conclusion follows from the premises, but if the conclusion is not believable, the algorithm actually checks whether the syllogism is valid. Note that the logically correct answer is guaranteed in case of any unbelievable syllogism. Also, note that the bias here occurs *before* any logical reasoning.

The algorithm of the selective scrutiny model determines the function ${ believable}$ function:

$$\begin{aligned} {believable}({{ If \,\phi _1 \, and \, \phi _2, \,then \,\psi }}, {{\mathcal {M}}}) = \; \; \; \; \; \; \; \; \; \; \; \; \; \; \; \; \; \; \; \; \; \; \; \; \; \\ \; \; \; \; \; \; \; \; \; \; \left\{ \begin{array}{ll} { true,} & \text{ if } \psi \text{ holds } \text{ in } {{\mathcal {M}}} \\ { valid}({{If\,\, \phi _1 \,\,and \,\,\phi _2, \,\,then\,\, \psi }}), & \text{ otherwise } \end{array} \right. \end{aligned}$$

As described, the algorithm determines a ${ believable}$ function, but not vice versa, different algorithms can give rise to the same function.

**Fig. 2:**
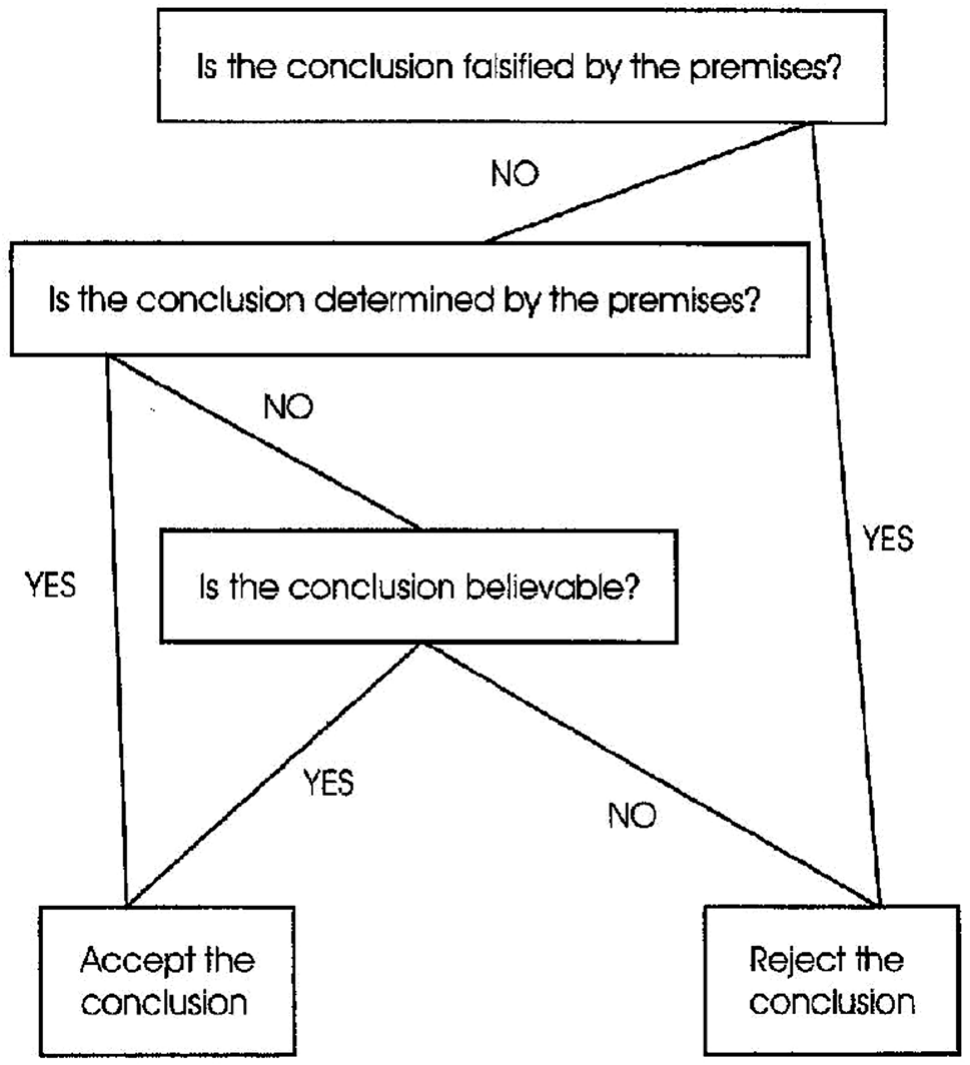
The misinterpreted necessity model, taken from Klauer et al. (2000)

**Fig. 3:**
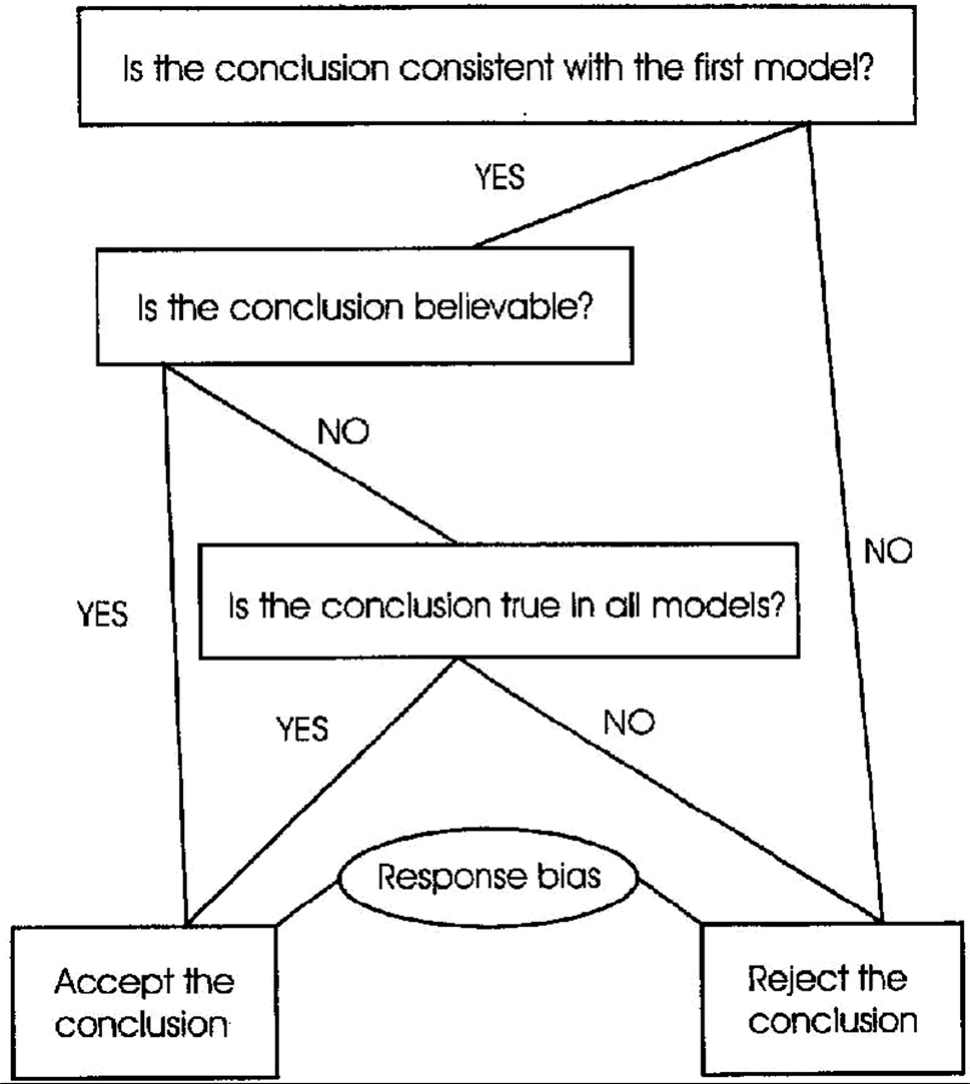
An account by mental models, taken from Klauer et al. (2000)

A more complicated algorithm is what is called the *misinterpreted necessity model*, which is described by the flowchart-like diagram in Fig. 2. This model involves a distinction between two kinds of invalid syllogisms, cf. Klauer et al. (2000), page 853: A syllogism is called *determinately invalid* if the conclusion is falsified by *all* models of the premises, whereas it is called *indeterminately invalid* if the conclusion is falsified by *some* models of the premises and verified by others. A feature of this algorithm is that the logically correct answer is guaranteed if the conclusion follows from the premises or if the conclusion is falsified by the premises (in the latter case the syllogism is determinately invalid). If none of these two conditions are satisfied, that is, if some models of the premises falsify the conclusion and some models verify it (the syllogism is indeterminately invalid), then the output of the algorithm is decided by the conclusion’s believability. Thus, the logically correct answer is guaranteed for any syllogism that either is valid or determinately invalid. Note that the bias here takes effect *after* the logical reasoning process.

Like in the case of the selective scrutiny model, it is straightforward to see that the misinterpreted necessity model determines a ${ believable}$ function, we leave the details to the reader.

According to Klauer et al. (2000), the bias in the misinterpreted necessity model is due to the subject’s misunderstanding of what it means to say that a conclusion does not follow from the premises, namely that it is sufficient that the conclusion is falsified by *some* models of the premises, not necessarily *all* such models.

Earlier we discussed the invalid “rose” and “mice” syllogisms, which have exactly the same logical structure, cf. Table 1. Since syllogisms with this structure have models of the premises that verify the conclusion (the “rose” case) as well as models that falsify it (the “mice” case), they are indeterminately invalid. Thus, in these syllogisms the response of the misinterpreted necessity model is decided by the believability of the conclusion, so in the “rose” syllogism, the response would incorrectly be “valid”but in the “mice” syllogism, the response would correctly be “invalid" (but for the wrong reason).

The paper Klauer et al. (2000) also gives an account of belief bias in syllogistic reasoning based on the “mental models” school in the psychology of reasoning, according to which the mechanism underlying human reasoning is the construction of models, (Johnson-Laird 2008). An account by mental models is shown in Fig. 3. The first step of this algorithm is to build an initial model of the premises of the syllogism under investigation, which is followed by an evaluation of the conclusion in the model in question. If the conclusion comes out as true, but it is not believable, this triggers the generation of further models of the premises, as indicated in the figure. Note that like in the misinterpreted necessity model, the logically correct answer is guaranteed for any syllogism that either is valid or determinately invalid. But if a syllogism is indeterminately invalid, then the answer becomes incorrect if and only if the conclusion is true in the initial model and also believable, hence, the selection of initial model matters. Note that the bias here takes effect *during* the reasoning process.

The mental models account of Klauer et al. (2000) leaves open how an initial model is produced from the premises, but if a function reflecting such a procedure is provided, it is straightforward that a ${ believable}$ function is determined.

Above we considered the mental model analysis of syllogistic reasoning given in Klauer et al. (2000), but many other such analyses can be found, see the references in Khemlani and Johnson-Laird (2012) and also the more recent example Bischofberger and Ragni (2020). However, most of the mental model studies of syllogistic reasoning deviate from the scenario we consider in the present paper: (i) In many studies, the experimental subjects are given the two premises, but not the conclusion, and have to figure out which conclusions follow from the premises. (ii) The algorithmic models of many studies do not involve information about the real world, hence, they cannot model belief bias.

## The conjunction task

Next we analyze the performances in the conjunction task called the Linda task, investigated in Morsanyi et al. (2010). Once again, the task is analyzed on both computational and algorithmic levels. Below we provide a brief discussion on the empirical study as reported in Morsanyi et al. (2010).

**An empirical study by** Morsanyi et al. (2010): The conjunction fallacy in Morsanyi et al. (2010) included tasks where a description of a person was given followed by a set of options where the experimental subjects were asked to rank these options in order of likelihood. This involves a fundamental law of probability theory, saying that the probability of two events occurring together (in “conjunction”) cannot be higher than the probability of either of the events alone. One example is what is called the Linda test, originally put forward by the psychologists Amos Tversky and Daniel Kahneman in Tversky and Kahneman (1983). The subjects read a description of Linda, a 31 years old, single, outspoken, and very bright woman. She majored in philosophy. As a student, she was deeply concerned with issues of discrimination and social justice, and also participated in anti-nuclear demonstrations. When asked to judge a number of statements about Linda according to how likely they were, most subjects ranked the statement “Linda is a bank teller and is active in the feminist movement” above the statement “Linda is a bank teller,” thus committing the conjunction fallacy.

Another test with the same structure involves a short description of a 10-year-old kid named Tim followed by certain statements about him, cf. Morsanyi et al. (2010), page 1381. The participants were asked to rank these statements in order of likelihood on a scale of 1–4, with one being most likely and 4 being least. Both the ASD group and the control group were found to commit the conjunction fallacy where they ranked the conjunction option, “Tim has a rabbit and he often plays football” more likely than the single option, “Tim has a rabbit.” However, the conjunction fallacy was significantly lower in the ASD group ($z = -1.97; p <.05$) making them less susceptible to the conjunction fallacy than matched controls.

This suggests that individuals with ASD are less influenced by contextual factors in their reasoning and might have a weaker influence of the task context (the provided description) while rating the conjunctive response options. Here the conjunction law is the norm which suggests that context/description should not affect the ranking of the statements.

The paper Morsanyi et al. (2010) explains this result in terms of what is called the Weak Central Coherence (WCC) theory of autism, cf. Happé and Frith (2006). According to this theory, people with autism tend to perceive parts rather than wholes, that is, they tend to process pieces of information in isolation, in comparison to typically developing people, that automatically process information in a more holistic way. The paper Morsanyi et al. (2010), page 1385, formulates autistic people’s lack of automatic contextualization in a very concrete way: Contrary to the case where only one piece of information has to be integrated with a context, “...when the representations of the two statements have to be integrated with both each other, and a description, this process seems to break down in the case of autistic participants”.

### Computational level analysis (conjunction task)

Comparing the likelihood of two statements under a certain context can be modeled by a function ${ rank}$ taking three arguments, namely two statements and a context, and returning a partial ordering of the set containing the two statements and their conjunction. Such a function is analogous to a function ${ believable}$ that models a subject judging a syllogism (cf. Sect. 2.1). Context-independence of a ${ rank}$ function, similar to that of a believable function can be formulated as

3 $$\begin{aligned} {rank}(h_1,h_2,e_1)={ rank}(h_1,h_2,e_2) \end{aligned}$$

for any statements $h_1$, $h_2$ and contexts $e_1$, $e_2$. In addition, probabilistic correctness of a ${ rank}$ function can be formulated as

4 $$\begin{aligned}&(h_1 \wedge h_2, h_1) \in { rank}(h_1,h_2,e) \hbox { and }\nonumber \\&(h_1 \wedge h_2, h_2) \in { rank}(h_1,h_2,e) \end{aligned}$$

for any $h_1$, $h_2$, and *e*. Here $(h_1 \wedge h_2, h_1) \in { rank}(h_1,h_2,e)$ means that the partial order ${ rank}(h_1,h_2,e)$ orders $h_1 \wedge h_2$ less than or equal to $h_1$, that is, the likelihood of the statement $h_1 \wedge h_2$ is judged to be less than or equal to the likelihood of the statement $h_1$. This is similar to correctness of a ${ believable}$ function mentioned above.

Note that the probabilistic correctness requirement leaves open whether ${ rank}(h_1, h_2, e)$ orders $h_1$ and $h_2$. Note also that a probabilistically correct ${ rank}$ function is context-independent if it satisfies the extra requirement that for any $h_1$, $h_2$, and *e*, the partial order ${ rank}(h_1,h_2,e)$ is a minimal partial order such that (4) is satisfied. In particular, in that case ${ rank}(h_1,h_2,e)$ does not order $h_1$ and $h_2$.

For our formal analysis, we consider Bayesian confirmation theory, which suggests that most participants focus on the degree of confirmation (Sides et al. 2002) while making their decisions, i.e., they compare the degrees to which the evidence raises the probabilities of the different hypotheses. To explain why the fallacy occurs, we use justification analysis by Shogenji (2012), a variant of the confirmation analysis. According to this theory, the conjunction fallacy occurs when the degree of justification for the conjunction is higher than the degree of justification for the conjunct i.e., $J(h_1 \wedge h_2,e) > J(h_1,e)$. Here *J*(*h*, *e*) is a measure of confirmation satisfying certain properties including the general conjunction requirement (GCR) (for details, see Shogenji (2012)).

The justification function can be used to define a particular rank function which provides intuitive reasoning as to why the fallacy might occur. This can be formulated as

5 $$\begin{aligned} {(h_1,h_2) \in rank}(h_1,h_2,e) \iff J(h_1,e) < J(h_2,e) \end{aligned}$$

for any $h_1$, $h_2$, and *e*. Here $h_1$ is judged less likely than $h_2$ if and only if $h_1$ seems less justifiable than $h_2$ given evidence *e*.

We can formalize the case where an individual commit the single conjunction fallacy by ranking the conjunctive statement “Linda is a feminist bank teller” higher than the single statement “Linda is a bank teller”.

6 $$\begin{aligned} \begin{array}{ccc} & ({ bank\_teller}, { bank\_teller} \wedge { feminist}) \in { rank}({ bank\_teller},{ feminist},e)\\ & \iff & \\ & J({ bank\_teller},e) < J({ bank\_teller} \wedge { feminist},e) & \\ \end{array} \end{aligned}$$

Thus, here the conjunction statement “Linda is a feminist bank teller” is ranked higher as it seems more justifiable given the evidence *e*, i.e., the description of Linda.

According to the analysis we described above, the fallacy occurs because people tend to use a cognitive process which is appropriate for choosing better-justified propositions even when that is not the task. The justification analysis is compatible with different theories of the cognitive process but what process an individual might use to determine the justification of a hypothesis depends largely on the context of the problem. For example, in Linda-like cases, the context of the problem, the description of Linda, is found to activate certain social stereotypes. Linda, a single 31-year-old woman who is active in discriminatory and social justice issues is more of a representative of the feminist movement than just a bank-teller. Similarly, from Morsanyi et al. (2010) we have that, Tim, living in a house with a garden, liking to play sports in the park, and collecting football cards, is more of a representative of the football-playing type rather than just having a rabbit. In such situations, individuals tend to automatically contextualise the input wherein they do not indulge in conscious, effortful reasoning. According to Shogenji (2012), whatever be the cognitive process used by individuals, the main aim is to use the process to choose propositions with higher degrees of justification.

### Algorithmic level analysis (conjunction task)

For the algorithmic explanation of the conjunction fallacy, once again, we delve deeper into the justification analysis as provided by Shogenji (2012). However, the first and foremost assumption that we make here is that *probabilistic reasoning* is a convincing way to analyze cognitive processes. For further discussion on this assumption, see Griffiths et al. (2012). According to this justification analysis of the conjunction fallacy, as explained above, evidence may justify the conjunction more than some conjunct. Even though such an explanation can be considered at a computational level, from the algorithmic level viewpoint this analysis by itself does not elucidate further. For this reason, we consider the probabilistic reasoning behind the justification analysis as given in Shogenji (2012).

Even though Shogenji—referring to Marr’s distinction—is “more interested in the *computation* (the input–output relation) that is accomplished than in the *algorithm* for the computation” (cf. Shogenji (2012), page 39), his formal analysis does constitute a sketch of the cognitive processes underlying the conjunction fallacy, in particular, he does not take the justification measure *J*(*h*, *e*) as an unanalysed and primitive entity, rather, he formally analyses the judgement in the conjunction fallacy $J(h_1 \wedge h_2, e) > J(h_1, e)$ in terms of the two probabilistic judgements: (i) $P(h_2 \vert e \wedge h_1) > P(h_2 \vert h_1)$ and (ii) $P(h_1 \vert e) < P(h_1)$ (cf. page 39 in Shogenji (2012)). Shogenji’s analysis is based on the corollary of Appendix 5 of Shogenji (2012), which says exactly that (i) and (ii) above implies $J(h_1 \wedge h_2, e) > J(h_1, e)$. An intuitive way to understand this mathematical result is to instantiate it to the statements about the conjunction task considered earlier, that is, the judgement

$$\begin{aligned} J({ bank\_teller} \wedge { feminist}, e) > J({ bank\_teller}, e) \end{aligned}$$

follows from the two probabilistic judgements

$$\begin{aligned} \begin{array}{cc} P({ feminist} \, \vert \, e \wedge { bank\_teller}) > P({ feminist} \, \vert \, { bank\_teller}) \\ \hbox { and }\\ P({ bank\_teller} \, \vert \, e) < P({ bank\_teller}) & \\ \end{array} \end{aligned}$$

This suggests that Shogenji takes this analysis in terms of probability theory as an account of the cognitive processes behind the fallacy.

A natural question arises here: How is the mathematical analysis of the fallacy $J(h_1 \wedge h_2, e) > J(h_1, e)$ in terms of (i) and (ii) more precisely involved in the cognitive processes? Is it involved in the cognitive processes like Newton’s laws are involved in our spatial cognition, even though our spatial cognition is more cognitively plausibly explained in terms of theories of the physical world that go under the heading “naive physics”? Whatever role this analysis exactly plays in the cognitive processes, we note that the equations (i) and (ii) involve the two statements $h_1$ and $h_2$, together with the context *e*, which fits the above quotation from Morsanyi et al. (2010), according to which it is the integration of two pieces of information and a context that “breaks down” for autistic people.

One way to understand the above mathematical result is that it shows the *robustness* of the analysis of the conjunction task in terms of justification, that is, the analysis does not directly depend on the actual choice of the justification measure *J*, rather, it boils down to the two conditions (i) and (ii) being satisfied, as discussed in Shogenji (2012). We should also mention a related result of Crupi et al. (2008), which analyses the fallacy in terms of Bayesian measures of confirmation, and shows that for many such measures of confirmation (denote them by c, say), if (i) $c(h_1, e) \le 0$ and (ii) $c(h_2, e \vert h_1) > 0$, then $c(h_1 \wedge h_2, e) > c(h_1, e)$. We note that in probabilistic terms, $c(h_1, e) \le 0$ implies that $P(e \vert h_1) \le P(e \vert \lnot h_1)$, and $c(h_2, e \vert h_1) > 0$ is equivalent to $P(e \vert h_1 \wedge h_2) > P(e \vert h_1)$.

## The decision task

We now analyze the performances in a decision task of choosing between pairs of consumer products in the presence of a third less desirable decoy product, investigated in Farmer et al. (2017). We analyze the task on computational as well as algorithmic levels.

**An empirical study by** Farmer et al. (2017): It is investigated whether individuals with ASD show reduced sensitivity to contextual stimuli when exposed to a decision-making situation where they had to make choices between pairs of consumer products that are presented with a third, less desirable decoy option. In a choice set, a decoy option is usually considered as an asymmetrically dominated alternative which is dominated by one of the choice alternatives but not by the other, i.e., based on the preference determining attributes, it is completely dominated by (i.e., inferior to) one option (target) and only partially dominated by the other (competitor). The choice task included participants to see 10 pairs of products (e.g., USB sticks); the products in each pair differed on two dimensions (in the case of USB sticks, storage capacity, and longevity). Each pair was presented twice, once with a decoy that targeted one product and once with a decoy that targeted the other. According to the conventional economic theory, any rational individual when exposed to such a situation should show a consistent preference behavior as the individual’s preference between two items should be independent of the ‘decoy’ options on offer. In contrast, it was observed that the choices of the general participants (control group) were heavily influenced by the composition of the choice set. Rather than being based on an independent assessment, the attractiveness of a given option relied upon how the individual compared it with the other values that were simultaneously present (attraction effect). But this tendency was quite reduced for individuals with ASD. Thus, they showed reduced sensitivity to contextual stimuli, indicating that their choices were more consistent and conventionally rational. In general, the individuals with ASD made fewer context-induced preference reversals making them ‘rational decision-makers’.

### Computational level analysis (decision task)

The reduced context effect in people with ASD might be a manifestation of their reduced understanding of, or concern for, the likely beliefs and appraisals of others. Thus, the choices of individuals with ASD have a better chance to satisfy the norm given by (9) than typical individuals (Baron-Cohen 2000).

In theory, the rational decision-makers are expected not to show sensitivity to context stimuli and be more consistent in their choices when they had to make choices in the situation mentioned above in the presence of a decoy option. Choice consistency should be the norm in this case. More formally, a given subject’s decision in a context can be modeled by a “choice” function which returns the chosen item from the finite tuple of possible choices, and the requirement for context-independence is given by:

9 $$\begin{aligned} \begin{array}{l} { Choice} ({ Product}_1, { Product}_2, { Decoy}_1) = \\ { Choice} ({ Product}_1, { Product}_2, { Decoy}_2) \end{array} \end{aligned}$$

Note that this is analogous to the requirement on the judgements of syllogisms that we called context-independence (requirement (1) on a ${ believable}$ function). On the other hand, there is no requirement similar to the correctness of the ${ believable}$ function (requirement (2) on the function).

### Algorithmic level analysis (decision task)

We now provide an algorithmic explanation of the attraction effect bias that is visible in context-dependent decision tasks (Farmer et al. 2017). To this end, we consider dimensional weight models as discussed in Wedell (1991); Ariely and Wallsten (1995), where the authors mention how the difference in dimensional (attribute) weights are highly dependent on the similarity relationship among the items. The more similar a set of items is on one attribute the easier it is to notice discrepancies on their other attribute (for both target and decoy items) so that the observed discrepancies on a given dimension increase the corresponding weight (Ariely and Wallsten 1995). Thus, once the decision-maker (DM) is able to determine the important dimension it then goes on to compare the three items (target, decoy, and competitor) on that dimension. After the comparison, the DM gives more attention weight to the target and decoy as the distance between them is smaller compared to that between competitor and decoy, eventually selecting the target as the final choice. This idea of giving higher attention weights to options whose attribute values are similar is based on the multiattribute linear ballistic accumulator (MLBA) model given by Trueblood et al. (2014).

**Fig. 4:**
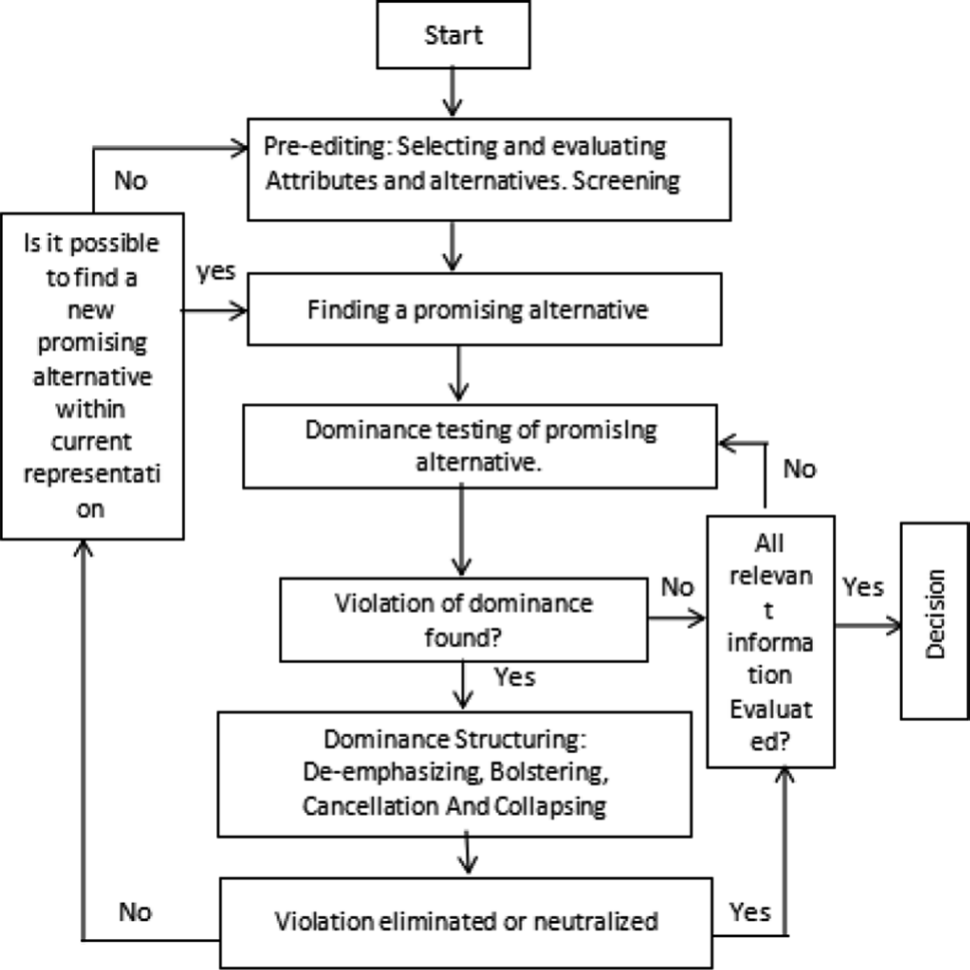
The decision process, adapted from Montgomery (1983)

**Fig. 5:**
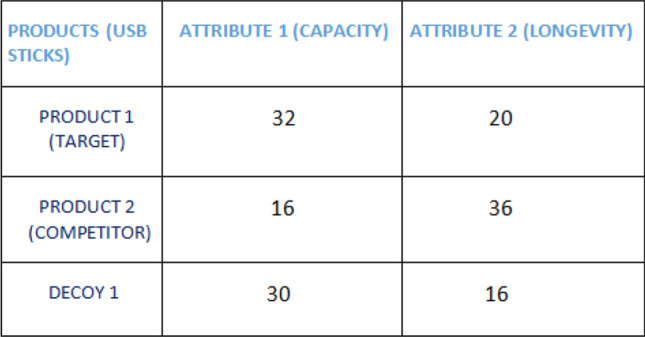
Slightly changed numerical example, adapted from Farmer et al. (2017)

The Dominance Search Model (DSM) of Decision Making (Montgomery 1983), which considers four phases of a decision process (cf. Fig. 4), is used to describe the decision process discussed above. We analyze the dimensional weight theory using the flowchart-like diagram in Fig. 4 and establish a line of argument as to how the decision task explained in Farmer et al. (2017) fits in this respect. However, this argument might vary with different examples especially in terms of given attribute values. The decision task in Farmer et al. (2017) considers a choice set with three items defined on two dimensions where the target strictly dominates the decoy. According to the Dominance Search Model, the DM follows four phases of the decision process:

*[Phase 1] Pre-editing: selecting and evaluating attributes and alternatives. Screening.* In the first phase, the DM screens and evaluates the attributes and alternatives. Alternatives with a better chance of becoming dominant are selected. In the decision task above the target strictly dominates the decoy thus reducing its chance of getting selected. This has been illustrated using Fig. 5 where the target completely dominates the decoy on both the attributes of capacity and longevity. The target attribute values, 32 and 20 are greater than decoy attribute values of 30 and 16. However, the DM might keep the discarded decoy in mind for the later stages of the decision process to check whether one alternative indeed dominates others. Thus in this phase, the DM considers target and competitor as acceptable alternatives and moves on to the next phase.

*[Phase 2] Finding a promising alternative.* Given the selected alternatives from the first phase, the DM now moves on to detect an alternative with attractive attributes that can be considered as a promising alternative (see Fig. 4). The bias becomes evident in this phase as the target shows a higher potential of being a promising alternative because of its strict dominance over the decoy. These phases imply a certain directionality of the decision process–some alternatives are given more attention in the later phases. For our case, the DM is unable to compare the target and the competitor and hence is unable to attend to the information supporting only one of them, although the target appears more attractive than competitor. As we can see from Fig. 5, the target is better than the competitor on attribute 1, the value 32 is greater than 16, but the competitor is better than the target on attribute 2, 36 is greater than 20, making it impossible to consider target as the final choice in this phase.

*[Phase 3] Dominance testing of promising alternative.* Once the DM is able to find a potentially promising alternative, the dominance test is done in this phase. If there is any violation, the DM caters to it in the next phase. In our case the target is considered to be a more promising alternative because of the attraction effect, and as a result, has some advantages in relation to the other alternatives. It now becomes pertinent to find whether there are any disadvantages of the promising alternative. The DM makes a comparative evaluation and tries to determine a dominance structure. A strict dominance of the target over the decoy is established but the target and the competitor are found to be incomparable as mentioned in the last two phases. This leads to a violation of dominance which the DM caters to in the next phase. If no violation is found, the DM checks whether all the relevant information has been evaluated (Fig. 4). Once this is done, the final decision is taken, otherwise, the DM moves on to test dominance once again (see Fig. 4).

*[Phase 4] Dominance structuring: de-emphasizing, bolstering, cancellation and collapsing.* After identifying a violation of dominance the DM tries to neutralize it in this phase using the ways mentioned in Fig. 4. Out of the four operations mentioned in Fig. 4, de-emphasizing is the most relevant one in our case. The DM attempts to de-emphasize the difference across alternatives on certain attributes using the idea of dimensional weights discussed above. While trying to structure dominance of target over competitor, the DM takes into consideration the decoy option once again, which was discarded earlier. First, the DM establishes the similarity between target and decoy on certain aspects and assigns higher weightage to the dimension on which they vary. Once that important dimension is established, attention weights are assigned based on that dimension while making comparison between target-decoy and competitor-decoy. This process could be explained using Fig. 5, where we observe that the target and decoy are almost similar in attribute 1, i.e., they have nearly identical attribute values: 30 and 32. As a result, the second attribute becomes more prominent to the decision-maker, leading them to assign a higher weightage to attribute 2. Since the decision-maker identifies attribute 2 as the important dimension, they proceed to compare the target-decoy and competitor-decoy on that dimension. Based on the MLBA mode discussed above, we observe that the distance between the target and decoy based on attribute 2 is smaller, i.e., the difference between their attribute values is small: 20 and 16, compared to the competitor and decoy. Consequently, larger attention weights are given to the comparison between the target and decoy, leading the decision-maker to select the target as the final choice. Psychologically, the attraction effect occurs because the target and decoy are more difficult to discriminate than competitor and decoy which leads to increased attention to target and decoy during the evaluation process thus making the target more attractive and leading the DM to select it at the end of the process. After possible removal of the violation, the DM moves on to make the final decision. Otherwise, the evaluation process starts again. We note that the decision process of Fig. 4 is task-dependent and might vary accordingly.

For the example discussed at the beginning of this section, it is seen that the target strictly dominates the decoy thus reducing its chance of getting selected, but both the target and the competitor are considered as options at this stage [Phase 1]. Now, the target is considered as the more promising alternative because of the attraction effect [Phase 2]. A strict dominance of the target over the decoy is established but the target and the competitor are found to be incomparable [Phase 3]. This leads to a violation that gets resolved in the next phase [Phase 4] which can be explained using the theories mentioned above.

We note here that the syllogisms discussed in Sect. 2 are endowed with a notion of (in)correct reasoning and bias in the algorithmic models amounts to various ways of deviating from this norm. In the case of the decision task, one can also consider norms, but we leave it to future work to investigate how the algorithmic models can capture such deviations from the relevant norms, if any.

## The sunk cost task

We finally analyze the performances in the sunk cost task, investigated in Fujino et al. (2019). As earlier, we analyze the task on computational as well as algorithmic levels.

**An empirical study by** Fujino et al. (2019): The sunk cost task has the following form: The subject is initially asked which travel destination he/she prefers out of two, for example, New York and Los Angeles. Then, further information is provided—the subject has mistakenly bought non-refundable tickets to both destinations, and the subject now has to choose between the destinations, which either have the same price (the control condition) or different prices (the sunk cost condition) where the initially non-preferred destination is 50% more expensive than the preferred destination. According to conventional economical theory, a rational decision maker should not pay attention to the price of the tickets since they have already been paid, only future consequences should be taken into account, so in the sunk cost condition, the subject should stick to the initially preferred destination.

A subject was exposed to a number of sunk cost trials as well as a number of control trials, and the susceptibility to the sunk cost effect was measured as follows: The percentage of the sunk cost trials where the initially non-preferred (but more expensive) destination was chosen minus the percentage of the control trials where the initially non-preferred (and equally expensive) destination was chosen. The study (Fujino et al. 2019) reports a number of results, in particular that the ASD group had a lower sunk cost effect score than a control group with matching individuals ($p < 0.01$).

The conclusions of the paper Fujino et al. (2019) are corroborated by the study Rogge (2021), having a different research design and more experimental subjects.

### Computational level analysis (sunk cost task)

Choice consistency is the norm, that is, choices have to be consistent in the sense that they should be the same, whatever be the past expenses. Formally, we can consider a choice function which returns the chosen item from the possible choices, where a third parameter in the function provides information about the phase of the task: The initial phase where the subject is asked about his/her preference or the second phase where full information has been disclosed, and the subject is asked where he/she wants to go. In our example, choice and preference are equal in the initial phase but they may not be the same in second phase due to the sunk cost effect.

The requirement for context-independence is then given by:

10 $$\begin{aligned} \begin{array}{l} { Choice} ({ Product}_1, { Product}_2, { InitialPhase}) = \\ { Choice} ({ Product}_1, { Product}_2, { SecondPhase}) \end{array} \end{aligned}$$

Of course, this is analogous to the choice function in connection with the decision task in Subsect, 4.1, but in that case the third parameter in the function provides information about a possible decoy product. So in Subsect. 4.1, the third parameter is a product that can actually be chosen by the subject (and the two decoys can be presented in either order), whereas in the sunk cost task, the third parameter provides information about the phase of the task (in this sense the sunk cost task is less symmetric, that is, it involves a temporal aspect).

As in the decision task of Subsect. 4.1, the requirement (10) is analogous to the requirement (1) on a ${ believable}$ function in the syllogistic task of Subsect. 2.1, but again, there is no requirement similar to correctness (2) of the ${ believable}$ function.

To understand the notion of correctness we dig deeper into the concept of preference. Economists usually equate preferences with choice or willingness to pay wherein they assume that the preferences of rational individuals are revealed through their choices. Such preferences are usually considered to be stable and inherent, i.e, they pre-exist and are not determined by context. However, research has shown many instances of preference fluctuations because of contextual influences such as option set, transient affect, framing of options, and others (Simonson 2008). This led to the notion that preferences are typically constructed while inherent, predetermined preferences play only a limited role. The decision task in our example does not have any universally correct answer, as we saw in the case of syllogistic or Linda tasks. Correctness in sunk costs occurs when choice consistency holds.

In the first phase where the participants have to mention their preference between LA and NY, we cannot exactly say whether this revealed preference is a function of inherent preference or constructed preference, or both. For participants who have had directly experienced a trip to both LA and NY might have an inherent preference that is considered to be more stable and unambiguous. As mentioned in Simonson (2008), inherent preferences are likely to play an important role in revealed preferences for experienced objects.(e.g., a pillow, an iPod-like media player, Dutch licorice candy) or life situations. Thus, individuals with a stable inherent preference over LA and NY should prefer the same option in both phases. However, when they choose a different option in the second phase from what they preferred in the initial phase we say their choices to be context-dependent.

The preference function returns the preferred destination from the finite tuple of number of possible options based on one’s stable inherent preferences, the correctness is thus given by:

11 $$\begin{aligned} \begin{array}{l} { Choice} ({ Product}_1, { Product}_2, { Phase_i}) = \\ { Preferred} ({ Product}_1, { Product}_2, { Phase_i}) \end{array} \end{aligned}$$

where *i* = Initial Phase, Second Phase. (11) should hold such that Eq. 10 is satisfied i.e., for any individual the correctness holds when choices and preferences are equal in both phases. In other words, if (11) does not hold in the second phase i.e., preference is not equal to the choice made we can say the choice decision is context-dependent.

The correctness will hold when they make the same choice in both the phases i.e, it will be context-independent. In our example, we are concerned with the second phase as the choice and preference are always equal in the first but not in the second.

### Algorithmic level analysis (sunk cost task)

**Fig. 6:**
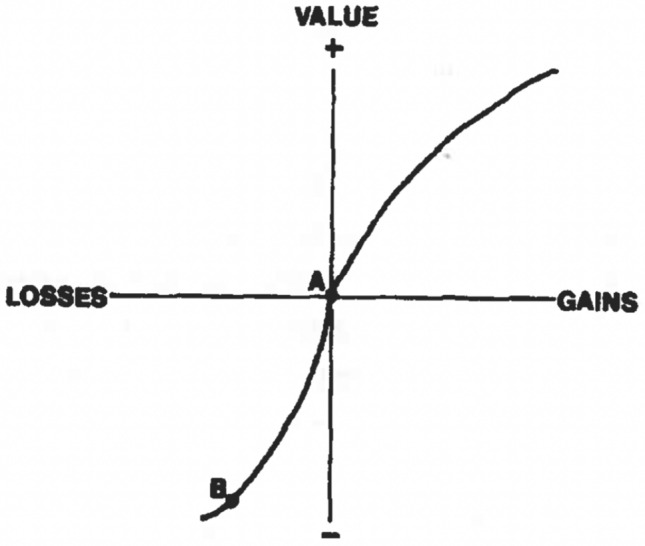
The value function of prospect theory, adapted from Arkes and Blumer (1985)

In this section, we algorithmically explain the decision process of individuals subjected to the sunk cost effect as discussed in Fujino et al. (2019). Many potential explanations have been provided for susceptibility to the sunk cost effect of which we explain our example using Thaler’s (Thaler 1980; Arkes and Blumer 1985; Thaler 1999) explanation of the sunk cost effect based on prospect theory (Kahneman and Tversky 2013; Tversky and Kahneman 1979). The theory talks about an individual’s psychic accounting system which includes perceiving outcomes as gains and losses, rather than as final states of wealth or welfare (Thaler 1980, 1999) According to prospect theory, gains and losses are defined relative to some neutral reference point which usually corresponds to the current asset position. They are represented by a value function (*v*) based on subjective decision weights rather than objective probabilities as seen in Expected Utility Theory (Kahneman and Tversky 2013; Tversky and Kahneman 1979). The essential characteristics of the value function are:

- It is defined over gains and losses with respect to some natural reference point, for reference see examples in Thaler (1980); Kahneman and Tversky (2013).
- It is concave for gains and convex for losses as seen in Fig. 6. The shape of the value function is based on the psychological principle that the difference between smaller values (say 0 and 100) seems greater than the difference between large values (say 1000 and 1100) irrespective of the sign of the magnitudes. This shape explains the risk seeking choices for losses and risk averse choices for gains. It is the shape of the value function that leads to the inconsistency in choices and preference reversals (cf. snowstorm example, Arkes and Blumer (1985), page 131).

Thaler considers gains and losses as pleasure and pain where pleasure is thought of as a value function in the domain of gains (*v*) and pain correspond to the value function in the domain of losses (${\tilde{v}}$). These value functions replace the utility functions as seen in Expected Utility Theory.

Using the four phases of the Dominance Search Model (DSM) (Montgomery 1983), as discussed previously in Subsect. 4.2, we analyze the above-mentioned theory and explain how individuals make their decisions when influenced by the sunk cost effect. The decision task in Fujino et al. (2019) includes two phases, the initial phase where no information is provided to the participants regarding the options while the second phase includes certain information. Both phases can be explained using the four phases as mentioned in Montgomery (1983). Before we move on to explain the decision process, it is important to mention that most laboratory experiments on decision making provide participants with alternatives having a well-defined attribute matrix as seen in Farmer et al. (2017), the example of USB sticks with two attributes. In such situations, the DM can determine the important attributes and acceptable alternatives in a clear cut manner with minimum constructive activity. However, in other situations like in our sunk cost example, the DM is confronted with ill-defined alternatives (only the choice options are mentioned and no other information provided in the initial phase) which implies that DM must actively search for or construct interesting attributes and alternatives to reach a final choice. Thus individuals attempt to create dominance by changing their representation of the decision situation such that one alternative becomes dominant. We first explain the Initial Phase using the DSM model (Montgomery 1983) and then move on to explain the second phase of the sunk cost task.

**THE INITIAL PHASE**: In this phase, the DM is presented with two travel destinations, New York (NY) and Los Angeles (LA), out of which they were instructed to select their preferred option.

- *[Phase 1] Pre-editing: selecting and evaluating attributes and alternatives. Screening.* The DM screens the alternatives based on certain attributes (weather, number of sightseeing spots, etc.) that they construct themselves, as the alternatives are not well-defined. In this phase, both NY and LA are considered acceptable alternatives.
- *[Phase 2] Finding a promising alternative.* In this phase one of the options (say NY) is considered a promising alternative the dominance of which is tested in the next phase. In the case of ill-defined alternatives, the selection of promising alternatives based on self-constructed attributes might depend on factors related to existing norms, habits, or other people’s opinions (cf. Montgomery (1983), page 356–358). For example, in our case, a DM might consider an option (NY) promising based on his or her preconceived notion which can be influenced by either of the factors mentioned above.
- *[Phase 3] Dominance testing of promising alternative.* Since no information is provided in the initial phase, the DM moves on to consider the promising alternative as the dominant option thus selecting it as the final choice.The DM does not come across any information that might lead to the violation of dominance for the promising alternative.
- *[Phase 4] Dominance structuring: de-emphasizing, bolstering, cancellation and collapsing.* The decision-maker (DM) does not reach the fourth phase, see Fig. 4.

**THE SECOND PHASE**: In this phase, the sunk cost condition is introduced for one group of participants where they are provided the following information:

1. The prices of the tickets are mentioned where the price of the nonpreferred city is 1.5 times more expensive than the price of the preferred city (mentioned in the initial/preference phase).
2. Participants are told that they have mistakenly purchased both the tickets and the prices paid for them are not refundable.
3. The departure date is the same for both the cities.

In presence of the sunk cost condition, the some participants were found to switch their choices (preferred city) in the second phase. In our explanation we consider NY to be the preferred city and LA the non-preferred one.

- *[Phase 1] Pre-editing: selecting and evaluating attributes and alternatives. Screening.*: Given the size of the choice set the DM considers both options to be acceptable and no option is discarded.
- *[Phase 2] Finding a promising alternative.* In this phase the DM considers LA (non-preferred city) to be a promising alternative because of the sunk cost effect. Based on the information that the DM has paid a higher non-refundable price for the LA ticket and also that they can choose only one option makes them consider LA as the desired option (loss aversion). Given that $p_{LA} > p_{NY}$ the DM now considers $v(g_{LA})>v(g_{NY})$. This mental accounting is due to susceptibility to the sunk cost effect.
- *[Phase 3] Dominance testing of promising alternative.* In the third phase the DM faces a violation of dominance as they still believe that the expected net pleasure from NY is more than that of LA, given their preference in their initial phase, thus making them unsure of LA as a potential choice. However, because the DMs are influenced by the sunk cost effect they try to neutralize this violation by structuring the dominance of LA over NY in the last phase.
- *[Phase 4] Dominance structuring: de-emphasizing, bolstering, cancellation and collapsing.* Here the DM perceives the options solely as losses rather than gains. According to Thaler (1980), when the DM is not able to use a ticket for which he/she has paid for, it makes the ticket value zero, incorporating a feel of loss of price (*p*), that is, ${\tilde{v}}$(-p). Since $p_{LA} > p_{NY}$ the DM bolsters the idea that the net gain from ‘traveling to LA’ would be higher than ‘traveling to NY’. This could be formally explained using the convexity of the loss function: Assuming $v(g_{LA})=-{\tilde{v}}(-p_{NY})$, we have: ${\tilde{v}}[-(p_{NY}+p_{LA})] > {\tilde{v}}(-p_{NY})+{\tilde{v}}(-p_{LA})$

This implies that:

$$v(g_{LA})+{\tilde{v}}[-(p_{NY}+p_{LA})] >{\tilde{v}}(-p_{NY})+{\tilde{v}}(-p_{LA})+v(g_{LA})$$

And so we have:

13 $$v(g_{LA})+{\tilde{v}}[-(p_{NY}+p_{LA})] > {\tilde{v}}(-p_{LA})$$

where $v(g_{LA})$ is the gain from traveling to LA and  $v(g_{LA})=-{\tilde{v}}(-p_{NY})$ suggests that the enjoyment of going to LA is equivalent to the cost of not going to NY which is nothing but the loss of price for the NY ticket. This is similar to the snowstorm example in Arkes and Blumer (1985), page 131, where the enjoyment (gain) of the game is equal to the cost of enduring a snowstorm. In our example, the cost of enduring the storm is analogous to an individual choosing not to go to New York even after having paid for it. Equation (13) demonstrates that the net gain for the DM from traveling to LA exceeds the cost of not going to LA, i.e., incurring the loss of the price for the LA tickets. The DM feels that the net gain from traveling to LA is higher than traveling to NY because of the sunk cost effect. They consider the loss of the LA ticket price to be greater than that of New York. Due to this bias, the DM makes LA their final choice based on the rationale of minimizing the loss and giving higher weightage to the net gain they receive from traveling to LA compared to NY.

Thus, due to the sunk cost effect, the some participants end up choosing the option for which they have paid a higher price (non-preferred city) even though the expected pleasure/gain for it is less compared to the other option (preferred city).

## Discussion

In the previous four sections of this paper we have presented several tasks in which empirical validations exist for the supposition that individuals with ASD perform better than typical individuals. Now, a natural question to ponder upon: How to gain more insight into such findings so as to find some common pattern in reasoning? Till now we have explored these tasks individually from the perspectives of Marr’s computational as well as algorithmic levels. In what follows, we analyze and compare these tasks at both these levels for a better understanding.

### Analysis and comparison at the computational level

To analyze and compare the tasks on the computational level, we took a functional approach, that is, we modeled a subject’s reasoning by a mathematical function, where the function considered in a task took arguments representing the stimulus of the task, including contextual information, which are mapped to possible responses. For such functions, we considered the following properties:

- contextual independence
- correctness

The syllogistic task and the conjunction task gave rise to similar functional expressions (as defined by the mathematical functions) pertaining to both the properties, where correctness corresponded to logical and probabilistic correctness, respectively. As expected, in both cases correctness implies contextual independence (in the case of probabilistic correctness, given a small extra requirement). Note that the syllogistic and the conjunction tasks share the feature that the correct judgement can be carried out without any knowledge about the context/world.

On the other hand, the functional expressions corresponding to the decoy task and the sunk cost task also paved the way for considering the context independence. The notion of contextual independence in these tasks differed from one another. In the decoy task the third parameter of the function was an item that can be chosen which led to contextual sensitivity. For the sunk cost task the contextual sensitivity emerges from the information that the phase of the task provides. The decoy task was based on certain attributes which are explicitly mentioned, and no single choice was a dominant one (i.e., strictly preferred over the others), hence no notion of correctness. One might argue that such a correctness condition may be added to the decision task in case one of the choices is a strictly dominant one. But, more often than not, these tasks have rather complex choices. The sunk cost task does not include options that are based on particular attributes, rather, individuals state their choices based on inherent preferences. The equality of the preferred function and the choice function captures the notion of correctness. Thus, in terms of the notion of correctness the decoy task and the sunk cost task show distinctive features. Coming back to the syllogistic tasks, the contextual information here was explored through the consideration of belief biases.

To summarize, the commonalities in these tasks on the computational level exist in terms of the effect of contextual stimuli, though the in-depth analyses of such contexts provide us with certain distinguishing features. See the overview in Table 2, note in particular the pairwise similarities between tasks.

**Table 2:**
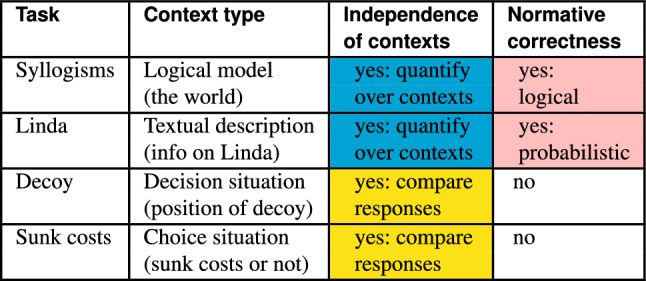
Summary of tasks where pairwise similarities between tasks indicated with colors (shades in the printed version)

### Analysis and comparison at the algorithmic level

From the algorithmic viewpoint, let us first make a comparison of the tasks in terms of their suitability for an algorithmic analysis. For the syllogistic task, the mental model analysis in Subsect. 2.2 shows how the subtleties of the belief biases can be elaborated on using an algorithmic framework. The conjunction task in Subsect. 3.2 was analysed in terms of Bayesian confirmation theory, more precisely, in terms of justification as suggested by Shogenji (2012). This analysis leaves open the query regarding how the probabilistic statements are precisely involved in the cognitive processes. There is no doubt in the suitability of the algorithmic analysis of the decoy task (cf. Subsect. 4.2) bringing out the attraction effect in the same. Lastly, for the sunk cost task the algorithm explains the sunk cost bias introduced in the second phase. Both decoy and sunk cost use the Dominance Search Model (DSM) to explain the decision process (cf. Subsect. 5.2).

At the algorithmic level, one question to ask is whether the origin of the bias in a biased reasoning algorithm can be clearly identified in the description of the algorithm, for example temporally, in the form of an identifiable stage of the algorithm where the reasoning goes wrong. This was possible in the case of the syllogistic task in an exemplary way: Here we considered three example algorithms (taken from Klauer et al. (2000), cf. Subsect. 2.2) where we could clearly pinpoint at which stage in the reasoning process the bias kicks in, namely respectively *before* (selective scrutiny), *during* (mental models) or *after* (misinterpreted necessity) the remaining reasoning process.

In the case of the conjunction task, it was not possible to identify the origin of the bias *in* the description of the algorithm, rather, the bias here was located at the meta-level, so to speak, in the sense that the biased experimental subject in the conjunction task replaces one task (the literally formulated question “which statement is most probable”) by another task (the question “which statement is best justifiable”). Of course, the context is irrelevant for the first question, but directly relevant for the second question.

The algorithm given by DSM for the decoy task includes two main effects which are as follows:

- attraction effect
- dominance effect

These biases consider the entrance of possible promising alternatives within the process itself, and as such, we have an ongoing process of introduction of the alternatives at different phases. We note that the corresponding notion of attraction effect is considered throughout the four phases of the decision making process considered according to DSM. In case of the sunk cost task these effects are not explicitly entailed in the algorithm given by DSM. Compared to the decoy task the alternatives presented to the participants in sunk cost are not well-defined where the attributes of each option are not clearly mentioned. The participants screen the alternatives based on self-constructed attributes which helps them determine the dominant option. In our example, the sunk cost bias enters only in the second phase when the sunk cost condition is introduced in the form of certain information provided to the participants. The attraction and dominance effect visible in the second phase is the result of the sunk cost bias.

Another question to ask is whether the output of a reasoning algorithm is uniquely determined by the input to the algorithm, or it is not completely determined by the description of the algorithm. The case of the syllogistic task is exemplary: Given a certain input to the selective scrutiny and misinterpreted necessity algorithms, the output is completely determined, however, the mental models account leaves open how an initial model is built from the premises of the input syllogism, thereby possibly affecting the output.^4^

In the case of the conjunction task, the bias is located at the meta-level, that is, bias amounts to a certain task (in terms of probability) being replaced by another one (in terms of justification), but when probability/justification is determined, the output of the algorithm is also is determined.

In the case of the decoy task, the bias occurs throughout the decision process. In DSM, the introduction of input (options with corresponding attribute values) in the pre-editing phase gives rise to the attraction effect bias, where the presence of the decoy option makes the targeted option more attractive because of its strict dominance over the decoy option. The dominant option is determined as the algorithm’s output. On the other hand, the sunk cost task has two phases with different inputs. The input in the second phase, the sunk cost condition in the form of additional information, gives rise to the sunk-cost bias. Similar to the decoy task, the output is determined by establishing the dominance of one option over another. However, unlike the decoy task, the options presented to participants as inputs in the sunk cost task do not have well-defined attribute matrix. Participants are presented with options with no straightforward attributes and attribute values. Some additional information are presented along with the choice options that allows individuals to search for or construct interesting attributes to reach a final choice. Thus even though the decoy and sunk cost task follow the same algorithm, the inputs vary in a sense that the way the options are presented to the participants are different.

So what can we express about which tasks, and what are the similarities and differences? When the tasks are analyzed at the abstract computational level, cf. the previous subsection, the tasks exhibit certain similarities as summed up in Table 2, but when they are analyzed at the more concrete algorithmic level, the differences are made explicit with respect to the handling of biases, as explicated in the present subsection.

### Further issues

The main objective of this work has been to determine the similarities and differences between the tasks at an abstract level. However, an important aspect worth mentioning is the homogeneity of the participant pool across the various studies considered here.^5^

All the empirical studies followed a treatment-to-control experimental design, except for the study conducted by Lewton et al. (2019); Lewton (2018). In their study, the treatment group included participants who either self-reported their diagnosis of ASD with written documentation, or were formally tested using specific criteria, such as those of the *DSM-V* manual,^6^, before participating in the experiments. Lewton’s (Lewton et al. 2019; Lewton 2018) experimental design included studying a general population based on obtaining the level of autistic traits using the AQ questionnaire, without any clinical diagnosis. Regardless of the measures undertaken, all the studies determined the level of autistic traits in participants. Additionally, all studies included some form of cognitive or intelligence tests to match samples between the treatment and control groups, and these tests also served as a control in determining participant performance in the tasks. A summary of the experimental designs can be found in Table 3.

**Table 3:** Summary of experimental designs

Task	Study	Type of study	Method of data collection	Other cognitive measures
Syllogisms	Lewton et al. (2019)	Observational study	Non-clinical adults recruited via web bulletins and ads at the University of Bath	Raven’s Advanced Progressive Matrices and Cognitive Reflection task
Linda	Morsanyi et al. (2010)	Treatment-to-control design	*Treatment group:* Adolescents from secondary schools in Plymouth *Control group:* Students from a secondary school in Plymouth	Short form of the Wechsler Intelligence Scale for Children
Decoy	Farmer et al. (2017)	Treatment-to-control design	*Treatment group:* Recruited through the University of Cambridge Autism Research Centre *Control group:* Recruited via the PureProfile platform	International Cognitive Ability Resource
Sunk costs	Fujino et al. (2019)	Treatment-to-control design	*Treatment group:* Recruited from volunteers with ASD at the outpatient units of the Showa University Karasuyama Hospital *Control group:* Recruited from the general population in the Tokyo area	Wechsler Adult Intelligence Scale or Japanese version of the National Adult Reading Test (JART)

The participant pool in these studies was generally homogeneous in terms of age, consisting of adults aged 18–71 years on average, except for the study by Morsanyi et al. (2010), which focused on an adolescent group. None of the studies found any significant age differences between the treatment and control groups. One of the main reasons is that the empirical studies by Farmer et al. (2017); Fujino et al. (2019); Morsanyi et al. (2010) included an experimental design where the participant samples were matched based on demographic factors as well as cognitive ability scores. In terms of gender composition, there was some variation in the female-to-male ratio, but no significant differences were found between the groups. Fujino’s study (Fujino et al. 2019) measured a few other demographic variables like education level and current smoking status, but no significant differences were observed based on these factors either. The participant pool in these studies came from developed countries like the US, UK, and Japan, which might suggest a certain homogeneity in the general population’s cognitive and intelligence tests. All participants with a higher level of autistic traits scored higher on cognitive and intelligence tests thus suggesting a positive correlation.

To conclude, considering all the demographic factors examined in the empirical studies, we find that the participant pools were mostly homogeneous with no significant differences between the groups.

One might ask whether there are emotional components in the tasks involved in the studies we consider.^7^ The paper Lewton et al. (2019) by Lewton et al.. makes some general remarks that we think are also of relevance to studies other than the syllogistic tasks mentioned there: According to Lewton et al. (2019), page 7, rather than applying rapid heuristics to deal with emotional issues, autistic individuals tend to apply more deliberative reasoning strategies. The paper Lewton et al. (2019) suggest that this tendency is parallel to applying a contemplative strategy when solving logical tasks, ignoring task-irrelevant contextual information.

However, to the best of our understanding, emotional behaviours tend to have a minimal influence in probabilistic tasks, e.g., conjunction fallacy, in the sense that the role of context becomes predominant, see Morsanyi et al. (2010). In addition, for the transactional and practical decision making tasks like those explained in Sections  4 and 5, cf. Farmer et al. (2017); Fujino et al. (2019), it might be arduous to comprehend the role of emotions.

## Conclusion and further work

In the present paper we consider four studies with reasoning tasks where ASD people perform better than typicals. Below we describe a further example study which we plan to subject to a closer analysis, but will leave for the future.

The study Martino et al. (2008) investigates adult’s performance on a financial task in which the monetary prospects were presented as either loss or gain, and it is shown that ASDs demonstrates a larger consistency in decision making than typicals.^8^ The paper explains this as a failure of ASDs to incorporate emotional cues into the decision process, thus, it is paradoxically the case that an impairment in processing contextual emotional information leads to a more consistent behavior in situations of risk. This study is replicated in Shah et al. (2016) with the same conclusion, that ASDs exhibit enhanced consistency.

The present study allows us to address the emerging discussion of whether autism is best understood in *medical* or *neurodiversity* terms, Baron-Cohen (2017). Here, proponents of the medical view argue that in highly social and unpredictable environments, some of the differences in individuals with autism may manifest as disabilities, while the neurodiversity view suggests that autism should rather be seen as variations different from the neurotypical brain. Thus, the neurodiversity view suggests that autism should rather be seen as a different cognitive style that should be valued as a strength in some contexts, where the environment should fit the person rather than vice versa. By identifying similarities and common features of the four tasks we have considered, in particular at Marr’s computational level, we believe that our study speaks in favor of the neurodiversity view.

From a practical and societal perspective, this line of work would be of help in psycho-educative terms, that is to understand the condition of ASD better, together with all the deficits and strengths associated with it. Moreover, mapping the cognitive strength of individuals with ASD will increase their employability, cf. Lorenz et al. (2017).

## Footnotes

1 Autism Spectrum Disorder is a psychiatric disorder with the following diagnostic criteria: 1. Persistent deficits in social communication and social interaction. 2. Restricted, repetitive patterns of behavior, interests, or activities. For details, see *Diagnostic and Statistical Manual of Mental Disorders, 5th Edition (DSM-V)* published by the American Psychiatric Association.

2 The recent paper (Rozenkrantz et al. 2021) asks a similar question, but from a different perspective, namely the perspective of neuroscience.

3 In particular, in Braüner (2014) it is demonstrated that two seemingly dissimilar reasoning tasks, namely two different versions of a false-belief task called the Smarties task, have exactly the same underlying logical structure. Similarly, in Braüner et al. (2020) it is demonstrated that four second-order false-belief tasks share a certain logical structure, but they are also distinct in a systematic way. We remark that such a strategy was also pursued in the book (Stenning and van Lambalgen 2008), where it was shown that a false-belief task and what is called the box task have a logical structure similar to a third task called the suppression task.

4 Note that we are here referring to the mental models account described in Subsect. 2.2 (taken from Klauer et al. (2000)). Completely deterministic mental models accounts of syllogistic reasoning can be found elsewhere, for example in the form of implementations, see (Khemlani and Johnson-Laird 2012).

5 Thanks to the reviewer for bringing up this issue.

6 *Diagnostic and Statistical Manual of Mental Disorders, 5th Edition* published by the American Psychiatric Association.

7 Thanks to the reviewer for directing our attention to this issue.

8 That is, the ASD subjects were less susceptible to what is called the framing effect, in which people make different choices to mathematically equivalent options, depending on how they are presented.
